# Effect of prehospital cervical spine immobilization on mortality and neurological outcome in trauma patients: an observational cohort study with propensity score matching based on the trauma registry of the German trauma society (TraumaRegister DGU®)

**DOI:** 10.1186/s13017-026-00731-w

**Published:** 2026-09-22

**Authors:** David Häske, Björn Hossfeld, Rolf Lefering, Uwe Schweigkofler, Jan-Philipp Stock, Davut Deniz Uzun, Christoph Wölfl, Dan Bieler, Committee on Emergency Medicine, Intensive Care and Trauma Management (Sektion NIS)of the German Trauma Society (DGU)

**Affiliations:** 1 Center for Public Health and Health Services Research, University Hospital Tübingen, Tübingen, Germany; 2 German Red Cross Emergency Medical Service, Reutlingen, Germany; 3 Department Anesthesiology, Intensive Care Medicine, Emergency Medicine and Pain Medicine, Armed Forces Hospital, Ulm, Germany; 4 Institute for Research in Operative Medicine (IFOM), University Witten/Herdecke, Cologne, Germany; 5 Department for Trauma Surgery and Orthopedics, BG Unfallklinik Frankfurt, Frankfurt Am Main, Germany; 6 Department of Interdisciplinary Emergency Medicine, Klinikum am Steinenberg, Reutlingen, Germany; 7 Medical Faculty Heidelberg, Department of Anesthesiology, Heidelberg University, Heidelberg, Germany; 8 Department of Trauma Surgery and Orthopedics, Reconstructive Surgery, Hand and Plastic Surgery Musculoskeletal Center Neuwied, Neuwied, Germany; 9 Department for Trauma Surgery and Orthopedics, Reconstructive Surgery, Hand Surgery, Burn Medicine, German Armed Forces Central Hospital Koblenz, Koblenz, Germany

## Abstract

**Background:**

Prehospital cervical spine immobilization is standard trauma care intended to prevent secondary spinal cord injury, yet evidence for its effectiveness is limited, and adverse effects are increasingly recognized. Large registry-based comparisons accounting for injury severity are scarce. This study investigated the association between prehospital cervical spine immobilization and hospital mortality and neurological outcome in trauma patients.

**Methods:**

Retrospective, multicenter, observational cohort analysis of the TraumaRegister DGU® (2020–2022). Patients aged ≥ 1 year who were primarily admitted in Germany, Austria, or Switzerland and had documented cervical spine immobilization, pupil status, and prehospital Glasgow Coma Scale (GCS) were included. A propensity score from logistic regression on injury severity, mechanism, and prehospital parameters was used for exact 1:1 matching, repeated in the subgroup with cervical spine injury (AIS ≥ 2). Primary outcome: hospital mortality; secondary outcome: Glasgow Outcome Scale (GOS) at discharge from the acute trauma admission.

**Results:**

Out of 31,055 patients, the prevalence of cervical spine injury (AIS ≥ 3) was 3.5%, only 65.9% received cervical spine immobilization. Immobilized patients had higher injury severity and were more likely to have high-energy trauma or be treated by an emergency physician. In the matched cohort (n = 16,922), no clinically meaningful difference was observed in hospital mortality (12.0% with immobilization vs. 13.6% without; *p* = 0.001) or in crude neurological outcome at discharge as measured by the Glasgow Outcome Scale (GOS). In the subgroup with confirmed cervical spine injury (AIS ≥2; n = 1,362), mortality (17.9% non-immobilized vs. 18.2% immobilized, *p* = 0.888) and neurological recovery did not differ significantly between groups (vegetative state: 1.8% non-immobilized vs. 2.7% immobilized, good recovery 46.2% non-immobilized vs. 43.4% immobilized, *p *= 0.626). GCS change between scene and hospital arrival — a confounded, non-specific measure—showed marginally more improvement in immobilized patients (15.7% vs. 12.8%; *p *< 0.001).

**Conclusions:**

In this registry-based analysis, prehospital cervical spine immobilization—recorded as a binary variable—was not associated with a measurable difference in hospital mortality or in crude neurological outcome at discharge (GOS), even in patients with confirmed cervical spine injury. These findings do not support routine, undifferentiated immobilization.

## Introduction

Depending on the literature and the severity of trauma, 1 to 5 percent of trauma patients have a major spinal cord injury (SCI) [2, 3]. These patients are at risk for severe neurological consequences [4].

To prevent further damage during transport to the hospital, prehospital spinal immobilization has been recommended since the mid-twentieth century and has become standard practice in emergency medicine [1]. However, spinal injuries in particular are more frequently overlooked in the prehospital setting than other diagnoses, as Schweigkofler et al. were able to show [2].

For this reason, various partially validated decision tools are available for the indication of prehospital immobilization, such as the NEXUS criteria, the Canadian C-spine rule (CCSR), the Scandinavian recommendations, or the “Immobilization traffic light” (Immo-Ampel) [3–6].

Nevertheless, over time, there has been growing recognition of the disadvantages associated with immobilization, including general patient manipulation, pain, pressure ulcers, potential increases in intracranial pressure, and the extended time before hospitalization. These disadvantages contrast with the purported benefit of spinal immobilization in preventing secondary damage [7, 8]. However, there is limited data on secondary neurological deterioration, which has a prevalence of 0.025% [9]. In addition, the evidence for spinal immobilization is limited [10–13].

The lack of evidence and the resulting controversial discussion lead to individual interpretations, particularly in patient groups whose injury patterns are not primarily classified as polytrauma and in whom the indication for immobilization is less clear than in severely injured patients [3, 10, 12, 14, 15].

The recommendations for penetrating trauma appear more definitive in a risk assessment: Data from the TraumaRegister DGU® (TR-DGU) demonstrates high predictive value for spinal injuries in blunt trauma but not for penetrating trauma [16]. Similarly, Connell et al., in a retrospective analysis of more than 30,000 patients with a prevalence of 0.034%, advise against immobilizing alert patients with isolated penetrating trauma [17]. Haut et al. analyzed 45,284 patients with penetrating trauma, showing a mortality rate of 14.7% in the immobilized group versus 7.2% in the non-immobilized group (*p *< 0.001) [18]. Vanderlan et al. support these findings [19]. Consequently, multiple guidelines recommend avoiding immobilization in isolated penetrating trauma to prioritize time-critical surgical intervention [19–21].

Even in cases of blunt trauma, available studies have not demonstrated a clear benefit of routine immobilization [15, 19]. Specifically, no clear advantage of cervical collars could be demonstrated [1, 12].

The fundamental question is whether prehospital cervical spine immobilization confers a measurable clinical benefit across the heterogeneous trauma population.

### Goals of this investigation

This study aimed to investigate the effect of cervical spine immobilization in trauma patients on the primary outcome of mortality and the Glasgow Outcome Scale (GOS) as a secondary endpoint.

## Materials and methods

### Study design

A retrospective, multicenter, observational cohort study was conducted using routinely collected data from the TraumaRegister DGU® (TR-DGU).

### Database

The TR-DGU of the German Trauma Society [Deutsche Gesellschaft für Unfallchirurgie (DGU)] was founded in 1993. The aim of this multicenter database is to collect pseudonymized and standardized documentation of severely injured patients. Data are collected prospectively in four consecutive phases from the site of the accident until discharge from hospital: prehospital phase, emergency room and initial surgery phase, intensive care phase, and discharge phase. The documentation includes detailed information on demographics, injury patterns, comorbidities, pre- and in-hospital management, the course in the intensive care unit (ICU), relevant laboratory findings (including transfusion data), and the outcomes of each patient. The inclusion criteria comprised (i) hospital admission via an emergency room with subsequent ICU care; or (ii) vital signs upon hospital arrival and death prior to ICU admission.

The infrastructure for documentation, data management, and data analysis is provided by the AUC – Academy for Trauma Surgery (AUC; Akademie der Unfallchirurgie GmbH), a company affiliated with the German Trauma Society. The scientific leadership is provided by the Committee on Emergency Medicine, Intensive Care and Trauma Management (Sektion NIS) of the German Trauma Society. The participating hospitals submit their pseudonymized data to a central database via a web-based application. The participating hospitals are primarily located in Germany (90%); however, an increasing number of foreign hospitals also contribute data, including hospitals from Austria, Belgium, China, Finland, Luxembourg, Slovenia, Switzerland, The Netherlands, and the United Arab Emirates. Approximately 31,000 cases from over 700 hospitals are entered into the database annually. Participation in TR-DGU is voluntary. For hospitals associated with TraumaNetzwerk DGU®, however, the entry of at least a basic data set is obligatory for quality assurance reasons.

### Patient selection

**Fig. 1:**
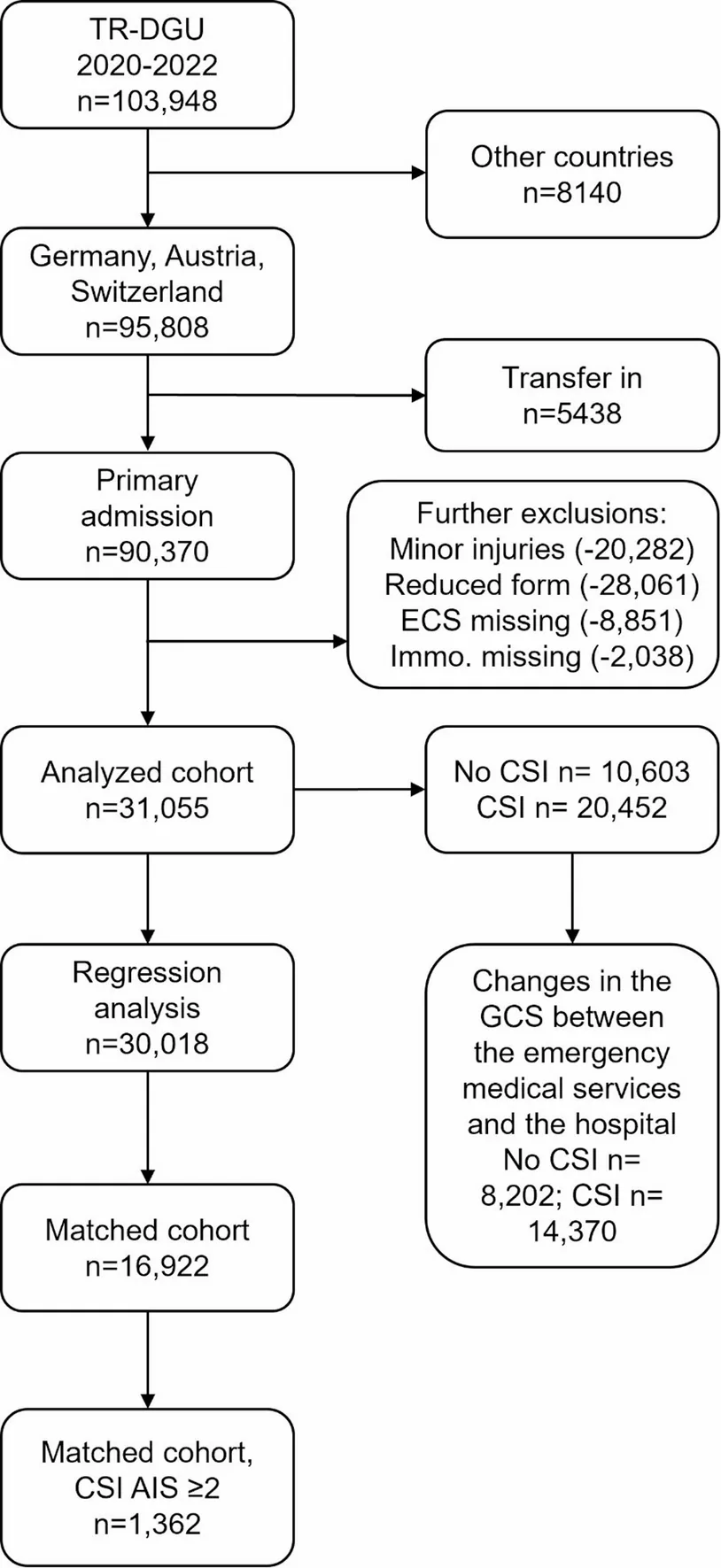
Patient flow diagram. Including regression analysis and a matched cohort based on that analysis, as well as a subgroup analysis of patients with cervical spine injuries (CSI) classified as AIS ≥ 2. In the other arm, the improvement, worsening, or no change in the difference in Glasgow Coma Scale (GCS) scores between the time of emergency medical service arrival and hospital admission was assessed

As the immobilization characteristic is only available in datasets from 2020 onwards, the available years 2020 to 2022 were included if they met the following criteria (Fig. 1):

- Data from Germany, Austria, and Switzerland
- Patients primarily treated at one of the participating hospitals, not transferred in from other hospitals
- Patient age ≥ 1 year
- Available data regarding cervical spine immobilization, pupil width, and light reaction, as well as prehospital Glasgow Coma Scale (GCS)

### Classifications

The Revised Injury Severity Classification, version II (RISC II) is used as a prognostic score [22].

#### Definition of cervical spine immobilization

In the TR-DGU, prehospital cervical spine immobilization is recorded only as a binary variable (yes/no); the specific technique applied (e.g., rigid collar, vacuum mattress, spine board, or manual in-line stabilization), the indication, and the quality of application are not captured.

#### Injury severity assessment

The Abbreviated Injury Scale (AIS) is an anatomical coding system that classifies and describes the severity of injuries across body regions and is widely used in trauma registries. Using AIS values, the Injury Severity Score (ISS) can be derived to quantify the overall severity of trauma. Patients are categorized as severely injured if they have an ISS ≥ 16. The abridged version adapted for the trauma registry was used for the analysis [23].

#### Motor function assessment

Neurological assessment was conducted using the Eppendorf–Cologne Scale (ECS) [24], a prognostic tool for traumatic brain injury (TBI) that incorporates the motor response, pupil reaction, and pupil size components of the Glasgow Coma Scale (GCS). The literature indicates that the ECS provides significantly greater accuracy in predicting TBI than the GCS [25]. For this analysis, only the motor response was utilized, categorized as normal (0 points), specific (1 point), non-specific (2 points), or absent (3 points).

#### Definition of polytrauma

Polytrauma is defined according to the Berlin definition, i.e., significant injury to three or more anatomical regions in two or more different anatomical AIS regions, in conjunction with one or more additional variables from the five physiological parameters [26].

### Statistical analysis

Statistical testing was used only for selected comparisons, as the large sample size would make even minor, non-relevant differences significant. Data are presented as means with standard deviations (SD) for continuous variables and as numbers of cases with percentages for categorical variables. For non-normally distributed data, medians/quartiles are presented instead of means. A propensity score for cervical spine immobilization was created using logistic regression with immobilization as the dependent variable. The propensity score derived from this analysis represents the probability of using cervical spine immobilization. The predictors included in this score are presented as odds ratios with 95% confidence intervals. The rounded propensity score was then used to identify pairs of patients with identical scores (exact matches), in which one patient received immobilization while the other did not. This matching was repeated in the subgroup of patients who had a cervical spine injury (AIS ≥2). The matched patient groups were compared using the chi-squared test for categorical variables and the Mann–Whitney U test for metric data. A p-value <0.05 was considered statistically significant. Statistical analysis was performed using SPSS version 29 (IBM Inc., Armonk, NY, USA).

## Results

During the study period from 2020 to 2022, 31,055 patients from 245 hospitals met the inclusion criteria. The mean age was 55 ± 22 years, and 70.5% (n = 21,908) of the patients were male. Immobilization was performed overall in 65.9% (n = 20,452) with varying frequencies in Austria at 43.7% (n = 1,067), Switzerland at 49.0% (n = 372), and Germany at 68.3% (n = 19,013). The hospital mortality rate was 13.6% (n = 1154) in the non-immobilization group and 12.0% (n = 1013) in the immobilization group.

**Fig. 2:**
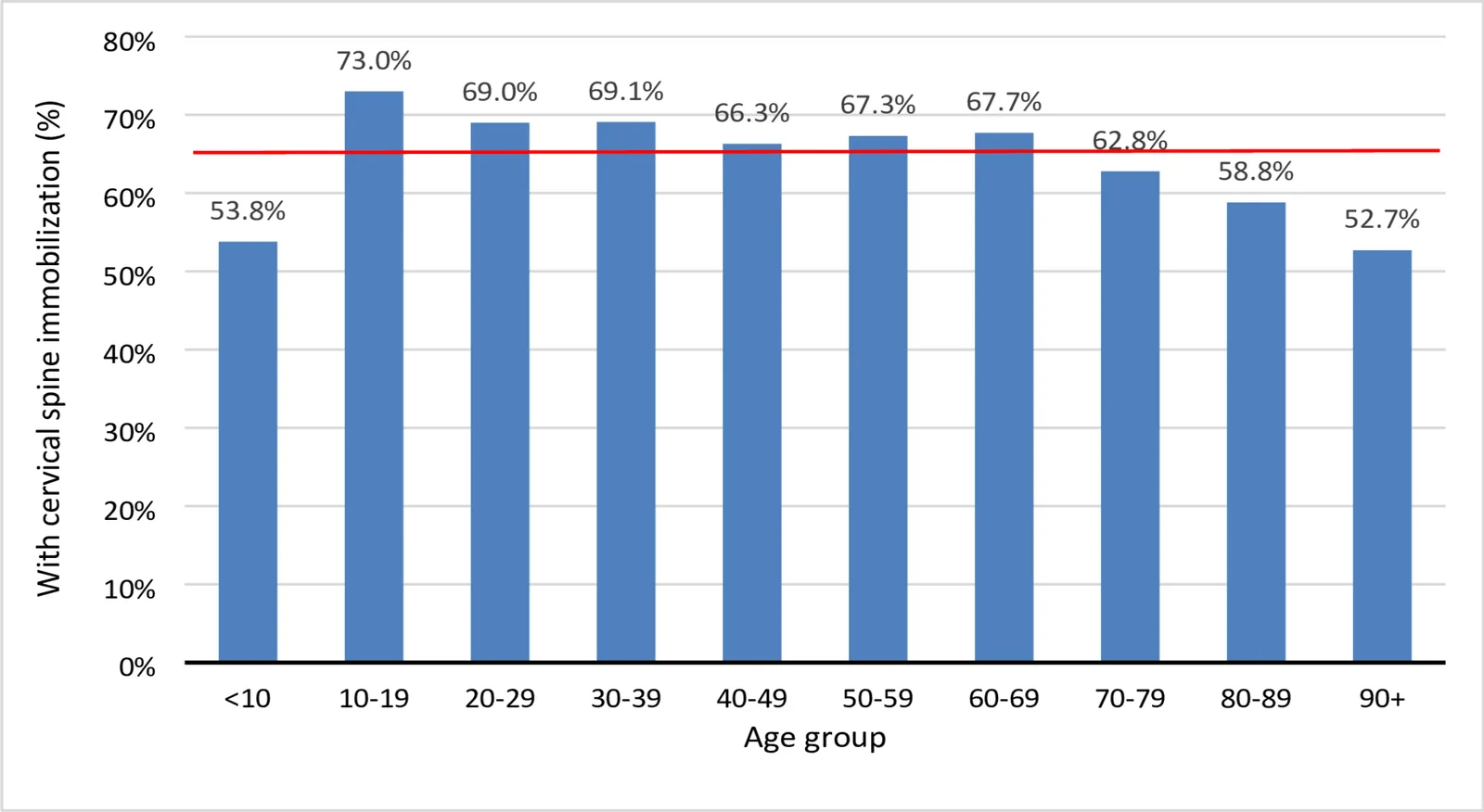
Rate of cervical spine immobilization in age groups; the red line indicates the overall average (65.9%)

**Fig. 3:**
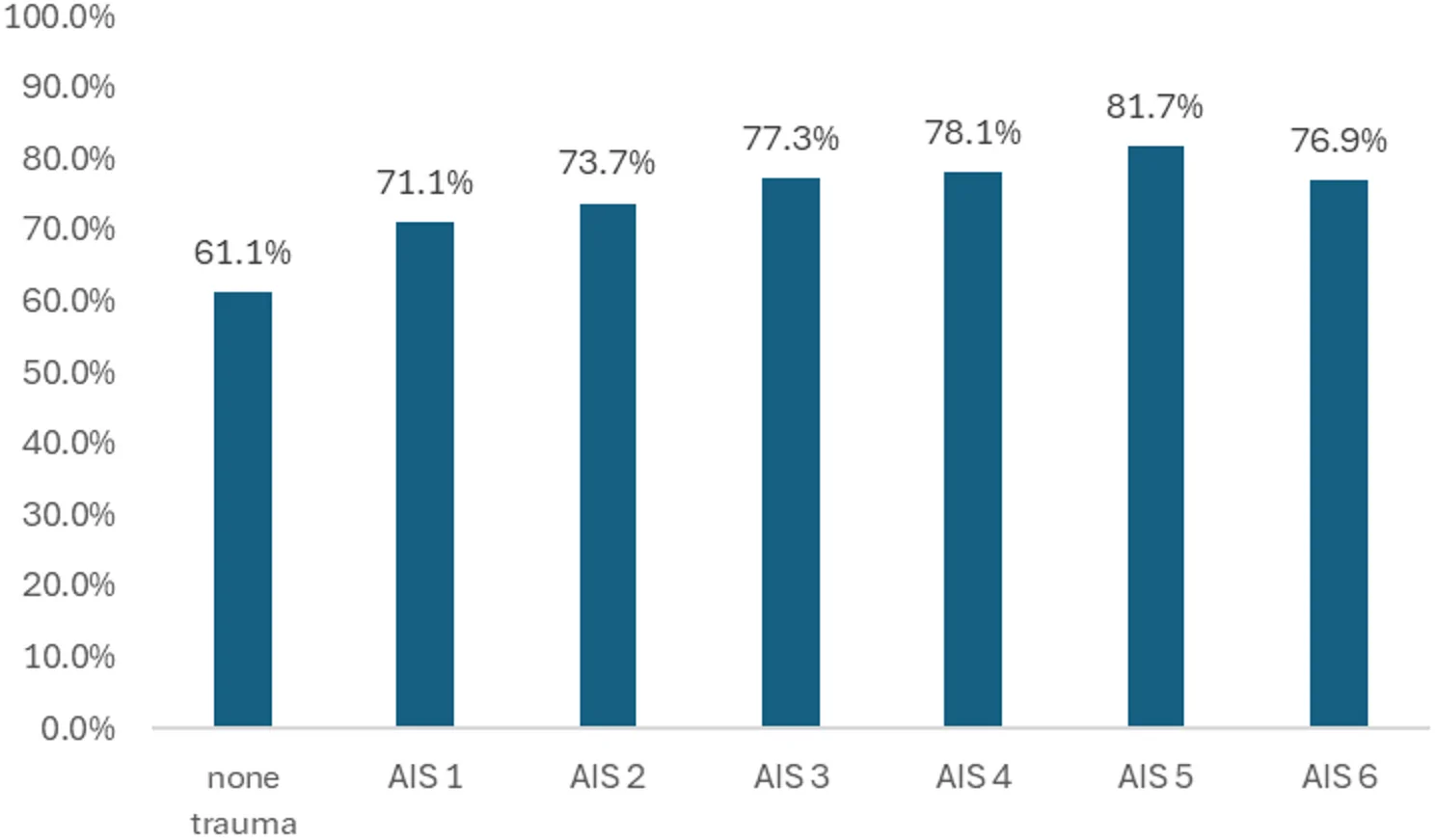
Proportion of immobilized patients by severity of spinal injury according to AIS in a post hoc analysis

The mean ISS was 18.1 ± 11.1 (immobilization group mean ISS 19.8 versus non-immobilization group mean ISS 17.5). However, AIS ≥3 cervical spine injuries account for only 3.5% of all patients (Table 1). The immobilization rate by age group is shown in Fig. 2. Figure 3 shows how the proportion of immobilized patients is distributed according to the severity of spinal injury as classified by the AIS.

**Table 1:** Descriptive analysis of patients with and without cervical spine immobilization with absolute and relative frequencies

	No cervical spine immobilization, n= 10,603	Cervical spine immobilization, n= 20,452
Age, mean (SD)	55.1 (22.8)	52.0 (22.0)
Females	3,189 (30.1%)	5,958 (29.1%)
Accident mechanism road traffic, car road traffic, motorbike road traffic, bicycle road traffic, pedestrian high fall (> 3 m) low fall (< 3 m) other	1,185 (11.5%) 878 (8.5%) 1,054 (10.2%) 421 (4.1%) 1,272 (12.4%) 3,244 (31.5%) 2,235 (21.7%)	3,850 (19.1%) 2,984 (14.8%) 2,732 (13.5%) 983 (4.9%) 3,955 (19.6%) 4,069 (20.2%) 1,610 (8.0%)
High energy trauma (car, motorbike, high fall)	3,335 (31.5)	10,789 (52.8)
Penetrating trauma	1,043 (9.8%)	289 (1.4%)
GCS ≤ 8 prehospital	1,551 (14.8%)	3,680 (18.1%)
GCS ≤ 13 prehospital	3,582 (33.8%)	7,877 (38.5%)
Motor function ECS prehospital 0 1 2 3	7,806 (73.6%) 1,553 (14.6%) 320 (3.0%) 924 (8.7%)	14,062 (68.8%) 3,341 (16.3%) 750 (3.7%) 2,299 (11.2%)
Injury Severity Score, mean (SD)	17.5 (10.8)	19.8 (12.1)
Polytrauma (Berlin definition)	1,372 (12.9%)	3,796 (18.6%)
Injuries (AIS ≥ 2) Head Thorax Abdomen Spine Pelvis Upper extremities Lower extremities	4,611 (43.5%) 4,400 (41.5%) 1,511 (14.3%) 2,496 (23.5%) 1,278 (12.1%) 2,839 (26.8%) 2,254 (21.3%)	9,950 (48.7%) 10,302 (50.4%) 3,091 (15.1%) 7,554 (36.9%) 3,693 (18.1%) 6,872 (33.6%) 5,294 (25.9%)
Prehospital shock (SBP ≤ 90 mmHg) Prehospital tracheal intubation Prehospital analgesia Prehospital fluid volume > 1000 ml	792 (17.8%) 1,883 (26.4%) 5,550 (52.3%) 780 (7.8%)	1,652 (8.7%) 5,246 (25.7%) 13,255 (64.9%) 2,358 (12.0%)
Prehospital Emergency Physician Treatment Prehospital Paramedic Treatment	8,986 (85.7%) 1,495 (14.3%)	19,153 (94.2%) 1,174 (5.8%)
Transport by ground EMS Transport by HEMS	7,910 (75.9%) 2,510 (24.1%)	13,380 (66.2%) 6,825 (33.8%)
On-scene time, minutes mean (SD) Hospital length of stay (days) median, quartiles	28.7 (14.6) 10 [5-19]	32.4 (16.7) 9 [4-17]

EMS, Emergency medical service; HEMS, Helicopter emergency medical service; ECS, Eppendorf-Cologne-Scale; GCS, Glasgow Coma Scale; SBP, Systolic Blood Pressure

By focusing on data sets with valid prehospital and hospital admission GCS, excluding patients with prehospital intubation, it can be determined whether GCS improves or worsens with or without cervical spine immobilization; GCS is a global measure of consciousness, not a sensorimotor or spinal cord outcome, and is heavily confounded by traumatic brain injury, analgesia, sedation, physiology, and airway management. The analysis showed a slight difference in GCS, but did not demonstrate a direct effect of immobilization on a secondary spinal cord injury (Table 2).

**Table 2:** GCS prehospital and GCS at hospital admission without intubated patients, and the difference between them categorized as unchanged, worsening or improvement by at least one point

	No cervical spine immobilization, n= 8,202	Cervical spine immobilization, n= 14,370	*p*-value
GCS, prehospital, median (IQR)	15 (15–14)	15 (15–14)	< 0.001
GCS, hospital admission, median (IQR)	15 (15–14)	15 (15–14)	0.33
GCS improved, % (n)	12.8% (1050)	15.7% (2255)	< 0.001
GCS unchanged, % (n)	72.4% (5940)	70.4% (10120)	
GCS deteriorates, % (n)	14.8% (1212)	13.9% (1995)	

GCS, Glasgow Coma Scale; IQR, Interquartile range

In the descriptive analysis, the low immobilization rate is striking, with 55.6% for low falls < 3 m, 21.7% for penetrating trauma, but also GCS ≤ 8 prehospital of 70.3%, prehospital motor function ECS=3 at 71.3%, head injuries 70%, prehospital emergency physician treatment 68.1%, prehospital paramedic treatment 44.0%, as well as relevant differences between air and helicopter.

### Factors influencing the application of cervical spinal immobilization

The multivariate logistic regression analysis included cervical spine immobilization as the dependent variable, based on a sample of n=30,018 patients (Table 3).

**Table 3:** Multivariate logistic regression analysis based on n=30,018 patients, with cervical spine immobilization as dependent variable

Predictor	Coefficient (SE)	Odds ratio	95%-CI	*p*-value
Age ≥ 65 years	-0.136 (0.030)	0.872	0.823–0.925	< 0.001
Penetrating trauma	-1.424 (0.076)	0.241	0.207–0.280	< 0.001
Prehospital GCS ≤ 13	0.395 (0.030)	1.485	1.401–1.574	< 0.001
Spine injury AIS ≥2	0.469 (0.030)	1.598	1.508–1.694	< 0.001
Prehospital tracheal intubation	0.231 (0.036)	1.260	1.175–1.351	< 0.001
HEMS Transport	0.198 (0.031)	1.219	1.147–1.296	< 0.001
Emergency physician at scene	0.749 (0.045)	2.115	1.937–2.308	< 0.001
Accident mechanism (reference: low fall)				< 0.001
road traffic, car	1.208 (0.051)	3.347	3.027–3.702	
road traffic, Motorcycle	1.294 (0.055)	3.649	3.276–4.065	
road traffic, bicycle	1.026 (0.054)	2.790	2.511–3.100	
road traffic, pedestrian	0.916 (0.071)	2.499	2.173–2.875	
high fall (> 3 m)	1.038 (0.051)	2.822	2.554–3.118	
Other	0.317 (0.048)	1.373	1.250–1.508	

Nagelkerke’s r^2^= 0.160

### Propensity score matching

The pairs selected for propensity score matching (8,461 pairs) showed the following properties in Table 4.

**Table 4:** Description of the patients matched according to the propensity score (n=16,922)

	No cervical spine immobilization, n= 8,461	With cervical spine immobilization, n= 8,461	Standardized mean difference
Age, mean (SD)	55.7 (22.5)	54.5 (22.2)	0.054
Female	2,605 (30.8%)	2,548 (30.1%)	0.015
Accident mechanism road traffic, car road traffic, Motorcycle road traffic, bicycle road traffic, pedestrian high fall (> 3 m) low fall (< 3 m) other	1,097 (13.0%) 826 (9.8%) 982 (11.6%) 388 (4.6%) 1,171 (13.8%) 2,665 (31.5%) 1,332 (15.7%)	1,247 (14.7%) 961 (11.4%) 921 (10.9%) 334 (3.9%) 1,735 (20.5%) 2,552 (30.2%) 711 (8.4%)	0.049 0.048 0.022 0.035 0.178 0.028 0.226
Penetrating Trauma	193 (2.3%)	250 (3.0%)	0.044
Polytrauma (Berlin definition)	1,141 (13.5%)	1,223 (14.5%)	0.029
GCS ≤ 13 prehospital	2,913 (34.5%)	2,930 (34.7%)	0.004
Motor function ECS prehospital 0 (normal) 1 2 3	6161 (72.8%) 1284 (15.2%) 271 (3.2%) 745 (8.8%)	6157 (72.8%) 1280 (15.1%) 235 (3.0%) 771 (9.1%)	0 0.003 0.012 0.011
Injury Severity Score, mean (SD)	17.8 (10.9)	18.4 (11.2)	0.054
Injuries (AIS ≥ 2) Head Thorax Abdomen Spine Pelvis Lower extremities	3845 (45.4%) 3549 (41.9%) 1104 (13.0%) 2166 (25.6%) 1126 (13.3%) 1895 (22.4%)	3987 (47.1%) 3950 (46.7%) 1054 (12.5%) 3079 (36.4%) 1268 (15.0%) 1826 (21.6%)	0.034 0.097 0.015 0.235 0.049 0.019
Prehospital Shock SBP≤90 mmHg Prehospital tracheal intubation Prehospital analgesia Prehospital fluid volume > 1000 ml	546 (6.9%) 1669 (19.7%) 4781 (56.5%) 635 (7.5%)	565 (7.0%) 1667 (19.7%) 4808 (56.8%) 639 (7.6%)	0.004 0 0.006 0.004
Prehospital Emergency Physician Treatment	7521 (88.9%)	7546 (89.2%)	0.010
Transport by ground EMS Transport by HEMS	6321 (74.7%) 2140 (25.3%)	6306 (74.5%) 2155 (25.5%)	0.005
On-scene time, minutes mean ± SD Hospital length of stay (days)	29.1 ± 15.4 9 (4 – 17)	31.0 ± 15.7 10 (5 – 18)	0.112

Some of the *p*-values were small, or even <.001; in these cases the observed difference between the groups should be checked for clinical relevance, because the large sample size is responsible for the rather small *p*-values. The actual difference in metric values (age, ISS) is rather small, with a standardized mean difference <0.02. EMS, Emergency Medical Service, HEMS, Helicopter emergency medical service; ECS, Eppendorf-Cologne-Scale; GCS, Glasgow Coma Scale; SBP, Systolic Blood Pressure; SD, Standard Deviation

For the comparative analysis using propensity score matching, cervical spine injury (AIS ≥ 2) was included with 681 pairs (Table 5).

**Table 5:** Propensity score matching, cervical spine injury (AIS ≥2)

	No cervical spine immobilization N=681	Cervical spine immobilization N=681	Standardized mean difference
Age (years), mean (SD)	58.9 (21.9)	59.5 (21.6)	0.026
Females	217 (31.9%)	213 (31.3%)	0.013
Accident mechanism road traffic, car road traffic, Motorcycle road traffic, bicycle road traffic, pedestrian Fall > 3 m Fall < 3 m other	130 (19.1%) 62 (9.1%) 71 (10.4%) 21 (3.1%) 128 (18.8%) 204 (30.0%) 65 (9.5%)	117 (17.2%) 61 (9.0%) 92 (13.5%) 21 (3.1%) 116 (17.0%) 211 (31.0%) 63 (9.3%)	0.049 0.003 0.096 0 0.047 0.022 0.007
On-Scene Time (min), mean (SD)	31.6 (15.6)	34.4 (16.1)	0.177
Penetrating trauma	9 (1.3%)	11 (1.6%)	0.025
Injury Severity Score, mean (SD)	23.6 (14.4)	23.5 (13.9)	0.007
Polytrauma (Berlin definition)	169 (24.8%)	161 (23.6%)	0.028
GCS ≤ 13 prehospital	270 (39.7%)	264 (38.8%)	0.018
prehospital suspicion of TBI (GCS ≤ 13 or suspected diagnosis)	412 (60.5%)	407 (59.8%)	0.014
Relevant injuries (AIS ≥2) Head Thorax Abdomen Pelvis Extremities	344 (50.5%) 335 (49.2%) 77 (11.3%) 77 (11.3%) 114 (16.7%)	313 (46.0%) 324 (47.6%) 78 (11.5%) 86 (12.6%) 121 (17.8%)	0.090 0.032 0.006 0.040 0.029
Complete cervical spine paraplegia Incomplete cervical spine paraplegia	61 (9.0%) 48 (7.0%)	76 (11.2%) 58 (8.5%)	0.073 0.056
Motor function ECS prehospital 0 (normal) 1 2 3	455 (66.8%) 99 (14.5%) 22 (3.2%) 105 (15.4%)	466 (68.4%) 101 (14.8%) 15 (2.2%) 99 (14.5%)	0.034 0.008 0.062 0.025
Cervical spine injury AIS 2 AIS 3 AIS 4 AIS 5 AIS 6	445 (65.3%) 115 (16.9%) 36 (5.3%) 67 (9.8%) 18 (2.6%)	402 (59.0%) 122 (17.9%) 27 (4.0%) 115 (16.9%) 15 (2.2%)	0.130 0.026 0.062 0.210 0.026
Shock (SBP ≤ 90 mmHg)	76 (12.6%)	82 (13.0%)	0.012
Prehospital tracheal intubation	177 (26.0%)	190 (27.9%)	0.043
Prehospital analgesia	373 (54.8%)	412 (60.5%)	0.116
Prehospital fluid volume >1000 ml	67 (10.6%)	92 (14.0%)	0.104
Treatment by EMS physician	613 (90.0%)	622 (91.3%)	0.045
Transportation by HEMS	208 (30.5%)	224 (32.9%)	0.052
Prognosis (based on RISC-II)	17.0%	15.7%	0.035
Outcome			p-Value
Hospital mortality	122 (17.9%)	124 (18.2%)	0.888
Surviving patients: Glasgow Outcome Scale PVS (Persistent Vegetative State) Severe Disability Moderate Disability Good Recovery	10 (1.8%) 116 (21.0%) 171 (31.0%) 255 (46.2%)	15 (2.7%) 123 (22.3%) 174 (31.6%) 239 (43.4%)	0.626
Length of hospital stay, days. Median (IQR)	12 (6–20)	12 (6–22)	0.377

EMS, Emergency Medical Service; HEMS, Helicopter emergency medical service; ECS, Eppendorf-Cologne-Scale; GCS, Glasgow Coma Scale; IQR, Interquartile range; SBP, Systolic Blood Pressure; SD, Standard Deviation

## Discussion

This retrospective registry analysis of more than 31,000 trauma patients showed that prehospital cervical spine immobilization was performed in 65.9% of patients. In neither the overall cohort nor the AIS ≥ 2 cervical spine subgroup did immobilization show a measurable association with survival or neurological outcome (GOS) at discharge. Mortality and functional outcomes did not differ between immobilized and non-immobilized patients in the propensity score matching. The incidence of complete or incomplete cervical spine paraplegia also did not differ between the two groups. However, it was not known when this occurred—initially during prehospital care or during clinical therapy.

### Who receives prehospital cervical spine immobilization?

Several factors influence EMS personnel’s decision to perform cervical spine immobilization. In addition to high-impact trauma, patients with polytrauma were more likely to undergo immobilization, primarily based on injury patterns and abnormal vital signs. In contrast, isolated penetrating trauma was rarely applied with immobilization, which aligns with current clinical guidelines [16, 27]. Increased patient age also appears to influence the decision against immobilization, as shown in Fig. 2. The findings of other studies indicate that in older age, minor trauma following a fall plays a greater role than multiple trauma [16].

Despite the generally accepted standard of immobilization for severely injured patients, this was done in only 65.9% of cases in our data, a remarkably low figure. Country differences are also striking: While 68.3% of patients in Germany were immobilized, the proportion was 49.0% in Switzerland and only 43.7% in Austria. Although uniform guidelines, such as the S3 polytrauma guideline, and established training programs, such as ITLS (International Trauma Life Support) and PHTLS (Prehospital Trauma Life Support), are widespread across all three countries, several factors may explain this discrepancy. First, the small number of contributing hospitals from Austria (n = 2,442 patients) and Switzerland (n = 759) compared with Germany (n = 27,854) limits representativeness and may reflect single-center practice rather than national patterns. Second, because immobilization is recorded in the TR-DGU only as a binary variable, different local thresholds for what is documented as “immobilization” (e.g., rigid collar alone versus full spinal motion restriction with vacuum mattress) may contribute to apparent rate differences. Third, EMS structures differ despite comparable training: physician staffing, dispatch criteria, and the relative role of HEMS vary across the three countries, and more experienced or physician-led teams tend to apply immobilization more selectively. Finally, the international shift toward selective spinal motion restriction may have been adopted at different speeds across centers and countries.

The role of rescue teams is also important: Regression analysis shows that immobilization is significantly more common when EMS physicians are involved (68.1%) than when only paramedics are involved (44.0%). Within the group of EMS-physicians, HEMS physicians (73.1%) immobilize more frequently than ground-based EMS-physicians (62.8%). As physicians in HEMS are predominantly qualified specialists, this could indicate a link between higher qualifications and more consistent application of standardized trauma care [28]. However, it is important to note that paramedics, in particular, receive intensive training in immobilization techniques. In some cases, much more than EMS physicians receive as part of their traditional and emergency medical training. This discrepancy raises questions about the actual factors influencing the use of immobilization. It suggests that, in addition to formal qualifications, other factors, such as experience, team dynamics, or systemic conditions, may also play a role. Differences in the case mix among emergency medical services could also play a role: In the countries included in the study, paramedics are generally dispatched to patients with less severe injuries, whereas ground-based emergency physicians—and especially helicopter emergency physicians—are more frequently called to more severe cases involving polytrauma, which was a strong predictor of immobilization in our regression analysis.

In addition, evidence in scientific literature shows that although staff rate their immobilization skills as good and feel confident, they do not perform the actual immobilization adequately [29–31]. Immobilization itself takes time, as it is often not performed as a solitary measure, which is reflected in the longer on-scene times. In any case, different immobilization techniques also require different amounts of time [32].

### Do outcomes differ between immobilized and non-immobilized patients?

The primary outcome was hospital mortality. In the propensity-matched cohort (n=16,922), mortality was 12.0% with immobilization versus 13.6% without immobilization (p=0.001); given the large sample size, this statistically significant difference is unlikely to reflect a clinically meaningful effect. In the subgroup with confirmed cervical spine injury (AIS ≥ 2; 681 matched pairs), mortality was virtually identical (18.2% with vs. 17.9% without; *p *= 0.888). The secondary outcome, neurological status at discharge measured by the Glasgow Outcome Scale (GOS), also showed no relevant difference between groups in this subgroup (good recovery 43.4% vs. 46.2%; vegetative state 2.7% vs. 1.8%; *p *= 0.626).

As an exploratory marker, we also compared changes in the Glasgow Coma Scale (GCS) between scene and hospital admission. The GCS is not a sensorimotor or spinal cord outcome and is heavily confounded by traumatic brain injury, analgesia, sedation, physiology, and airway management; it cannot be interpreted as a marker of neurological protection from immobilization. Immobilized patients showed a 2.9% higher rate of GCS improvement, while deterioration occurred 0.9% more frequently in non-immobilized patients (Table 2). Given the indirect nature of this measure and residual confounding, these descriptive differences should not be read as evidence of clinical benefit or harm.

Against this background, the potential risks of cervical spine immobilization remain debated: it has been hypothesized that immobilization impairs sensorimotor function or reduces vigilance by increasing intracranial pressure. Our data neither confirm nor refute such effects.

### Prior evidence

These findings are consistent with the available literature, which has likewise failed to demonstrate a clear benefit of routine cervical spine immobilization. A Cochrane review found no randomized trials of spinal immobilization in trauma patients and could not determine its effect on mortality or neurological outcome [10]. Hauswald et al. compared blunt-trauma outcomes between an immobilizing (USA) and a non-immobilizing (Malaysia) EMS system and found no benefit, with a signal toward possible harm [11]. Though this comparison has acknowledged limitations of setting, case mix, and reporting. Stronger signals come from registry analyses of penetrating trauma, where Haut et al. and Vanderlan et al. observed increased mortality with prehospital immobilization — a finding not directly transferable to our predominantly blunt-trauma cohort but underscoring that routine application is not without risk [18, 19]. Reflecting this evidence base, current recommendations have shifted from routine full immobilization toward selective spinal motion restriction (SMR). However, SMR itself is not uniformly defined across guidelines—recommendations differ on the role of cervical collars, long boards used for transport versus extrication, and manual in-line stabilization [3, 6, 33].

### What this means for future selective immobilization protocols

Having described practice variation and the outcome findings, this section turns to the implications for a selective, symptom-guided protocol. In the emergency setting, the primary goal of immobilization is to prevent secondary neurological deterioration, with secondary goals of analgesia and minimizing swelling and bleeding. However, the evidence is limited and inconsistent; the few available studies report an extremely low prevalence of secondary neurological deterioration of 0.025% [9, 34].

In the practice of prehospital trauma care, immobilization plays a central role and is the subject of extensive discussion. However, according to the data of this study, severe cervical spine injuries (AIS ≥ 3 have a very low prevalence of 3.5% and are ultimately immobilized in only 65.9% of cases; the advantages and disadvantages of immobilization are not visible here due to the limitations of the registry study.

The complexity of the current evidence and the low benefit in cases of questionable injury would most likely require an individual indication for immobilization supported by other studies [35]. However, because spinal trauma and its possible indications are often over- or underestimated, there is often a desire to define a valid protocol. In vigilant patients, clinical symptoms such as pain, malalignment/instability, and sensorimotor symptoms may point the way [16]. The regression analysis shows that spinal injuries with AIS ≥ 2 are more likely to lead to immobilization. Since AIS 2 fractures are often painful, pain may indicate the need for immobilization, as suggested by Häske et al. in their decision-making tool for spine immobilization in emergency medicine [6]. Surgical indications such as neurological symptoms, instability, and malalignment could be considered, but again, the evidence at the time and the exact indication do not appear to be high [27, 36, 37].

However, the challenge of spinal immobilization is not only the biomechanical component but also the consideration of other injuries (e.g., traumatic brain injury) and the patient’s general condition, such as respiratory or circulatory instability, which overshadow the importance of immobilization due to a life-threatening condition. For this reason, clinical assessment by trained professionals is always required. Particular attention should be paid to the cervical spine. Current tools such as rigid cervical orthoses, spineboards, and vacuum mattresses are our current answers. In discussing the disadvantages, the advantages must certainly be weighed, which is becoming increasingly difficult. The German S3 polytrauma guideline currently recommends that “transport should be as gentle and pain-free as possible” [27]. This recommendation is echoed in a recent commentary on the guideline’s spine chapter, which likewise frames the preferred immobilization procedure—rather than the underlying indication—as the unresolved focus of ongoing debate [38]. Irrespective of the equipment, this may well be the declared goal. The development of EMS stretchers from a flat ironing board to an almost ergonomically shaped stretcher significantly restricts movement, so it is certainly debatable whether additional immobilization tools are necessary, given our study results. Biomechanical studies have already shown that standard stretchers with additional head immobilization are not inferior to other methods [35]. Contemporary terminology is shifting from “immobilization” toward “spinal motion restriction” to signal a more selective, less rigid approach. As noted above, however, SMR itself currently lacks a uniform operational definition; clear, consensus-based operational standards must therefore accompany a change in terminology if it is to translate into reproducible practice.

In its recently published *Prehospital Trauma Compendium*, the National Association of EMS Physicians (NAEMSP) notes that current evidence does not establish spinal immobilization or spinal motion restriction as the standard of care. Accordingly, the document advises considering a reduction in the use of cervical collars and restricting the use of backboards or vacuum mattresses to the period of active extrication [33].

### Limitations

The overall prevalence of severe cervical spine injury (AIS ≥ 3) in our cohort was only 3.5%, so a potential benefit of immobilization may only be detectable in a very small group of patients. Patients with the most severe injuries (AIS 5–6) could not be analyzed separately because of the very small sample size.

The study is based on retrospective registry data and does not allow for causal inference. Immobilization was documented as a binary registry variable without information on the specific technique used (e.g., rigid collar, manual in-line stabilization, vacuum mattress, spine board), the extrication method, the timing, the duration, or the quality of implementation, and does not distinguish spinal motion restriction from full immobilization, precluding inference about specific techniques. Patients who died at the scene or during transport before hospital admission are not captured in the TR-DGU; if such prehospital deaths occur more often in severely injured—and therefore more often immobilized—patients, this survivor bias would attenuate any observed effect of immobilization on in-hospital mortality. Secondary neurological deterioration was not directly recorded. The available endpoints—GOS at discharge from acute admission (no 3- or 6-month follow-up, which would be standard for spinal cord injury research) and GCS change between scene and hospital arrival (confounded by traumatic brain injury and prehospital interventions) —are indirect and not specific to spinal cord protection. The registry therefore cannot exclude a specific effect of immobilization on secondary spinal cord injury. The TR-DGU uses the adult GOS for all ages, including the small pediatric subgroup, and does not record the ASIA Impairment Scale or sensorimotor level—precluding pediatric- and spinal-cord-specific outcome stratification. Residual confounding by indication cannot be excluded despite propensity score matching: the decision to immobilize is driven by on-scene assessments (mechanism, visible injury, EMS team type and experience) that are only partially captured in registry variables, leaving unmeasured drivers of the immobilization decision potentially unbalanced between groups. A similar interpretive challenge has been described for other prehospital interventions such as pelvic circumferential compression devices [39]. Prospective studies are needed to provide greater clarity.

### Conclusion

In this large-scale registry analysis, prehospital cervical spine immobilization was not associated with a measurable difference in hospital mortality or in crude neurological outcome as captured by the available endpoints (GOS at discharge; GCS change). Because these endpoints do not directly measure secondary spinal cord injury, a specific neuroprotective effect cannot be excluded. Nonetheless, the present data do not support routine, undifferentiated application. Taken together with the low prevalence of severe cervical spine injury, the substantial proportion of trauma patients not immobilized in current practice, and the absence of measurable benefit in this analysis, these findings argue against continued reliance on routine, undifferentiated immobilization. Instead, a selective, symptom-adapted approach to spinal motion restriction is required, guided by validated clinical decision tools and the patient’s overall context. In prehospital trauma care, interventions addressing immediate threats to oxygenation, circulation, and intracranial physiology should remain the priority, while immobilization should be applied selectively according to clinical risk.

## Data Availability

Requests for access to the data should be directed to the TraumaRegister DGU® of the German Trauma Society via email at traumaregister@dgu-online.de. Data access is subject to approval by the registry’s scientific committee and is restricted to on-site use at the TraumaRegister DGU® data center in accordance with data protection regulations.
